# LuxR Solos in the Plant Endophyte *Kosakonia* sp. Strain KO348

**DOI:** 10.1128/AEM.00622-20

**Published:** 2020-06-17

**Authors:** Susan Mosquito, Xianfa Meng, Giulia Devescovi, Iris Bertani, Alexander M. Geller, Asaf Levy, Michael P. Myers, Cristina Bez, Sonia Covaceuszach, Vittorio Venturi

**Affiliations:** a International Centre for Genetic Engineering and Biotechnology, Trieste, Italy; b Department of Plant Pathology and Microbiology, The Robert H. Smith Faculty of Agriculture, Food, and Environment, The Hebrew University of Jerusalem, Rehovot, Israel; c Istituto di Cristallografia-CNR, Trieste Outstation, Trieste, Italy; University of Tartu

**Keywords:** LuxR solos, bacteria, cell-cell signaling, endophyte, gene regulation

## Abstract

Endophytes are microorganisms that live inside plants and are often beneficial for the host. *Kosakonia* is a novel bacterial genus that includes several species that are diazotrophic and plant associated. This study revealed two quorum sensing-related LuxR solos, designated LoxR and PsrR, in the plant endophyte *Kosakonia* sp. strain KO348. LoxR modeling and biochemical studies demonstrated that LoxR binds *N*-acyl homoserine lactones (AHLs) in a promiscuous way. PsrR, on the other hand, belongs to the subfamily of plant-associated-bacterium (PAB) LuxR solos that respond to plant compounds. Target promoter studies as well as modeling and phylogenetic comparisons suggest that PAB LuxR solos are likely to respond to different plant compounds. Finally, LoxR is involved in the regulation of T6SS and PsrR plays a role in root endosphere colonization.

**IMPORTANCE** Cell-cell signaling in bacteria allows a synchronized and coordinated behavior of a microbial community. LuxR solos represent a subfamily of proteins in proteobacteria which most commonly detect and respond to signals produced exogenously by other microbes or eukaryotic hosts. Here, we report that a plant-beneficial bacterial endophyte belonging to the novel genus of *Kosakonia* possesses two LuxR solos; one is involved in the detection of exogenous *N*-acyl homoserine lactone quorum sensing signals and the other in detecting a compound(s) produced by the host plant. These two *Kosakonia* LuxR solos are therefore most likely involved in interspecies and interkingdom signaling.

## Abstract (precis)

Cell-cell signaling in bacteria allows a synchronized and coordinated behavior of a microbial community. LuxR solos represent a subfamily of proteins in proteobacteria which most commonly detect and respond to signals produced exogenously by other microbes or eukaryotic hosts. Here, we report that a plant-beneficial bacterial endophyte belonging to the novel genus of *Kosakonia* possesses two LuxR solos; one is involved in the detection of exogenous *N*-acyl homoserine lactone quorum sensing signals and the other in detecting a compound(s) produced by the host plant. These two *Kosakonia* LuxR solos are therefore most likely involved in interspecies and interkingdom signaling.

## INTRODUCTION

Quorum sensing (QS) is a bacterial cell-cell signaling system that relies on small compound signals and modulates cooperative behaviors important for bacterial fitness and host interactions (1, 2). The most common QS system in Gram-negative proteobacteria is mediated by *N*-acylhomoserine lactone (AHL) signals. The archetypical AHL-QS system is composed by two genes: the *luxI* family gene encoding an AHL synthase and its cognate *luxR* family gene that encodes a transcriptional factor that detects and responds to the cognate AHL (3). LuxR family proteins are characterized by an autoinducer (AHL)-binding domain at the N terminus (4, 5) and a DNA-binding helix-turn-helix (HTH) domain at the C terminus (6, 7).

Analysis of different genomes of proteobacteria has revealed the widespread presence of uncoupled *luxR* genes, which lack a cognate *luxI* gene; these are called LuxR orphans or solos (8–11). Only a few LuxR solos have been studied and found to evidence a role in intraspecies, interspecies, and interkingdom communication (12–15). One of the best-studied LuxR solos is SdiA of the *Enterobacteriaceae* (12). SdiA of Escherichia coli and *Salmonella* can bind and be stabilized by an endogenous signal (16) and respond to exogenous AHLs produced by neighboring bacteria and is involved in virulence in part due to the regulation of transcription of the *rck* (resistance to killing) operon (17–19). QscR from Pseudomonas aeruginosa, on the other hand, is a LuxR solo which responds to the endogenously produced AHL signals by the LasI-LasR system, and its role is to extend the LasI/R regulon (14).

A subfamily of LuxR solos found exclusively in plant-associated bacteria (PAB) has evolved to respond to plant low-molecular-weight molecules, thus forming an interkingdom signaling circuit (20). The members of this subfamily have some substitutions among the highly conserved amino acids in the AHL-binding domain and regulate the adjacently located proline iminopeptidase (*pip*) gene. Some PAB LuxR solos have been shown to be involved in plant virulence in members of the *Xanthomonas* genus or in plant-beneficial interactions in several *Pseudomonas* isolates (21–24). Recently, a plant leaf macerate and a plant compound have been implicated to induce the activity of the LuxR PAB solo called PipR in an endophytic *Pseudomonas* isolate (25). In addition, several studies have reported that plants possess AHL mimics which interfere with QS LuxR proteins (26, 27).

*Kosakonia* is a novel genus first described 2013 (28); several of its members are diazotrophs and efficient plant colonizers with plant growth-promoting (PGP) properties (29). Most *Kosakonia* strains have been isolated from economically important crops like maize, rice, wheat, sweet potato, sugarcane, and cotton (30–33). This genus is gaining attention as the analysis of a few recently available genomes has shown interesting features that would support their plant-associated lifestyle and PGP properties (29).

*Kosakonia* sp. strain KO348 has been isolated from an Italian rice cultivar and shown to possess PGP properties (34). This *Kosakonia* strain is an efficient rice rhizoplane and root endosphere colonizer harboring a type VI secretion system (T6SS) involved in plant colonization (35). *Kosakonia* KO348 does not produce AHLs, and we report here that it possesses two LuxR solos; one is an SdiA homolog designated LoxR, while the other one, designated PsrR, belongs to the subfamily of PAB LuxR solos that responds to plant signals. Structure-based modeling, putative target gene promoter expression analysis, and *in planta* studies were performed. In addition, *loxR* has been expressed in recombinant form, purified, and shown to promiscuously bind to AHLs.

## RESULTS

### *Kosakonia* contains two LuxR solos.

**FIG 1:**
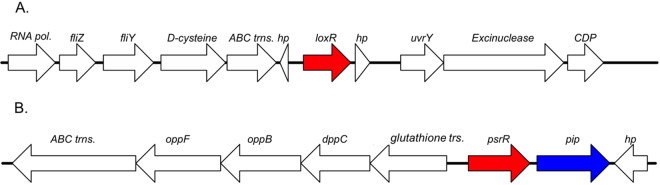
Gene maps of the two *luxR* solo loci present in KO348. (A) Ten-kilobase region of the genes surrounding the *loxR* gene. Genes are as follows: *RNA pol*., RNA polymerase sigma factor; *fliZ*, flagellar biosynthesis protein; *fliY*, periplasmic cysteine binding protein; *d-cysteine*, d-cysteine desulfhydrase; *ABC trns*., cysteine ABC transporter; *hp*, hypothetical protein; *uvrY*, response regulator; *Excinuclease*, excinuclease ABC subunit C; *CDP*, CDP-diacylglycerol-glycerol-3-phosphate 3-phosphatidyltransferase. (B) Eight-kilobase region of the surrounding loci of the *psrR* gene. Genes are as follows: *ABC trns*., dipeptide binding ABC transporter; *oppF*, oligopeptide transport ATP-binding protein; *oppB*, oligopeptide transport system permease protein; *dppC*, dipeptide transport system permease protein; *glutathione trs*., putative glutathione transporter; *pip*, proline iminopeptidase.

The genome of *Kosakonia* KO348 (36) possesses two *luxR* solo genes, and both have been annotated as single transcriptional units (Fig. 1). One LuxR solo, which we designated LoxR, displays its highest identity (76 to 99%) in its primary structure with SdiA from the *Enterobacteriaceae* family, while the other LuxR solo, which we designated PsrR, has its highest identity (71 to 99%) with a subfamily of PAB LuxR solos that respond to plant signals. Genetically adjacent to the *psrR* gene is the proline iminopeptidase (*pip*) gene, which is typical of this subfamily that responds to plant compound(s) (Fig. 1B). Both LoxR and PsrR have two PFAM domains conserved among the LuxR-type family proteins, the autoinducer binding domain (PF03472) and the bacterial regulatory protein, LuxR-type DNA-binding HTH domain (PF00196).

### LoxR is likely to respond to AHLs, whereas PsrR is likely to respond to a plant compound.

**FIG 2:**
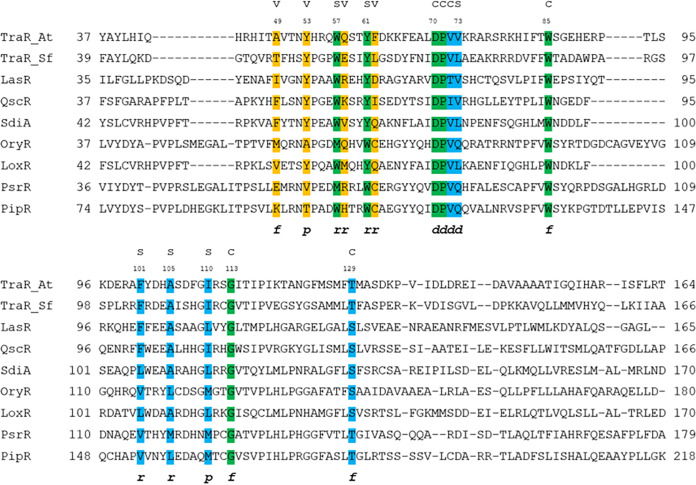
Structure-based multiple-sequence alignment of the regulatory domains of *Kosakonia* LuxR solos with members of canonical QS LuxR family and with the prototypes of the PAB LuxR solo subfamily. The residues belonging to clusters 1 to 3 are highlighted in green, cyan, and orange, respectively. The 3D architecture of the boundaries of the ligand-binding site is schematized by *r* (roof), *f* (floor), *p* (proximal wall), and *d* (distal wall) and its tripartite topology by c (conserved core), s (specificity patch), and v (variable patch). TraR_At represents *Agrobacterium tumefaciens* TraR, and TraR_Sf represents *Sinorhizobium fredii* TraR.

**FIG 3:**
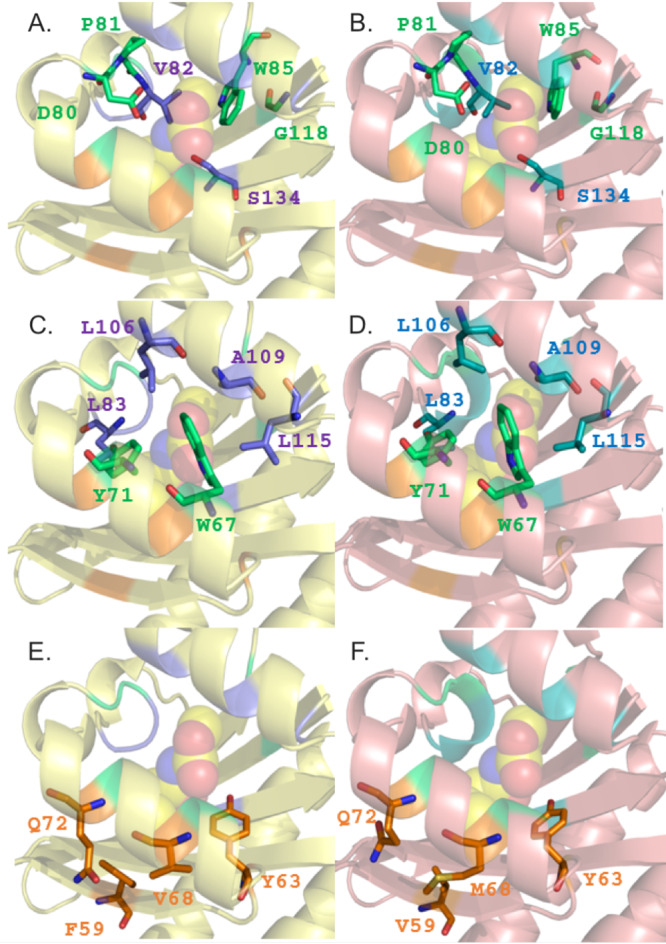
Comparison of the ligand-binding sites of LoxR with the QS LuxR solo SdiA. Shown are results of mapping of the protein residues defining the three clusters (colored with the same color code as used in Fig. 2) that delineate the conserved core (A and B), the specificity patch (C and D), and the variability patch (E and F) on the structure of SdiA in complex with 3OC_6_-HSL (PDB ID 4Y15) (16) (A, C, and E) and on the 3D structure-based homology model of the LuxR solo LoxR (B, D, and F). The carbon, nitrogen, and oxygen atoms of the 3OC_6_-HSL ligand shown in the left column are represented by spheres and are colored in yellow, blue, and red, respectively. Structures were produced by PyMOL (73).

**FIG 4:**
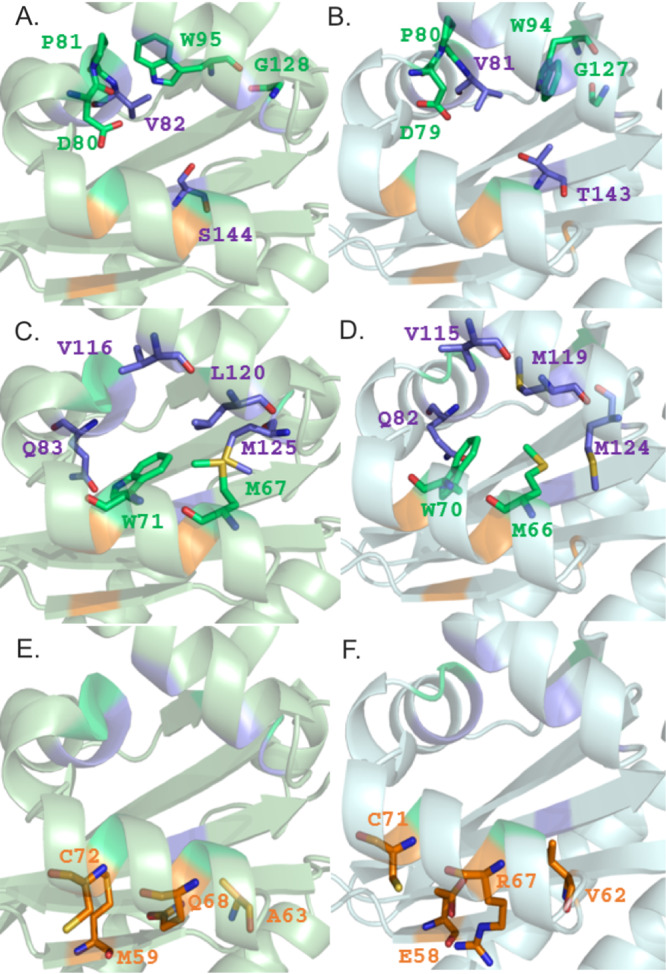
Comparison of the ligand-binding sites of PsrR with the prototype of LuxR PAB solo OryR. Shown are results of mapping of the protein residues defining the three clusters (colored with the same color code as used in Fig. 2) that delineate the conserved core (A and B), the specificity patch (C and D), and the variability patch (E and F) on 3D structure-based homology models of OryR (37) (A, C, and E) and of PsrR (B, D, and F). Structures were produced by PyMOL (73).

In order to gain insights into the structure underlying autoinducer specificity of LoxR and PsrR, we have resorted to multiple-structure-based sequence alignment and to structure-based homology modeling. In particular, we focused on the pocket residues directly interacting with the ligand that are conserved and belong to three previously described clusters (37). Cluster 1 comprises the six conserved hydrophobic/aromatic residues previously reported (3, 38), cluster 2 is composed of reasonably conserved residues directly involved in ligand binding (colored in green and cyan in Fig. 2 to 4), and cluster 3 consists of quite variable residues directly involved in ligand binding (variability patch colored in orange in Fig. 2 to 4).

This analysis revealed key differences between the ligand-binding pockets of LoxR and PsrR. We found in LoxR striking similarities to the structural determinants of the canonical QS LuxR family, while the ligand-binding site of the PsrR closely resembles the ones of PAB LuxR solo subfamily. In particular, not only has LoxR conserved the binding site core, including cluster 1 and 2 residues and delimiting the binding site floor and the distal wall, i.e., residues 70, 71, 72, 85, 113, and 129, according to *Agrobacterium tumefaciens* TraR (TraR_*At*) numbering (marked by “c” in Fig. 2), but also it has conserved all its residues of the specificity patch belonging to clusters 1 and 2 and mainly delimiting the binding site roof and the nearby proximal and distal walls, i.e., residues 57, 61, 73, 101, 105, and 110, according to TraR_*At* numbering (marked by “s” in Fig. 2). These differ from those in the subfamily of PAB LuxR solos and are conserved within the members of the canonical QS LuxR family, pinpointing that LoxR could have a common specificity toward AHLs. Residues belonging to both the conserved and the specificity patches in LoxR (Fig. 3B and D, respectively) are identical to those of the corresponding regions in the canonical QS LuxR solo SdiA (Fig. 3A and C, respectively). The crystal structure (PDB identifier [ID] 4LGW) of SdiA has been used as a template for this homology modeling since it displayed 68% and 83% overall primary sequence identity and homology, respectively. Moreover, some degree of conservation has been identified also in the respective variability patches (Fig. 3E and F) that comprise cluster 3 residues (delimiting the proximal wall and the nearby roof and of the floor of the binding site, i.e., residues 49, 53, 58, and 62 according to TraR_*At* numbering), usually less conserved even within the members of canonical QS LuxR family. Indeed, besides two identical residues (i.e., Y63 and Q72), a conservative substitution (i.e., M68 in LoxR instead of V68 in SdiA) and a semiconservative substitution (i.e., V59 in LoxR instead of F59 in SdiA) have been found. Overall, a close specificity of LoxR for AHLs can therefore be inferred.

The molecular determinants of the PsrR binding site (Fig. 4, right) suggest a different specificity. All the residues belonging to the specificity patch are conserved with respect to the PAB LuxR solo subfamily (Fig. 4, left) and differ with respect to the canonical QS LuxR family. Among the residues of the roof of the binding site, PsrR and OryR share the residue W71, which belongs to cluster 1 and is highly conserved among all members of the PAB LuxR solo subfamily. Conversely, in the corresponding position, the canonical QS LuxR family is characterized by the presence of the highly conserved residue Y61 (according to TraR_*At* numbering). Similarly, the two roof residues PsrR and OryR V115 and a conserved hydrophobic/aliphatic residue (L/M) in position 120 are replaced by the quite conserved TraR_*At* F101 and A105 residues, respectively. Among the residues of the distal wall of the binding site, PsrR and OryR Q83, which belongs to cluster 2 and is highly conserved in the PAB LuxR solo subfamily, is replaced in the canonical QS LuxR proteins by a conserved hydrophobic/aliphatic residue (V/L), TraR_*At* V73. Nevertheless, important differences can be detected in cluster 3 residues that are less conserved: the only position in this cluster that PsrR and OryR share is C71. Indeed, PsrR is characterized by the presence of two charged residues (E and R, instead of hydrophobic/aliphatic residue M and of hydrophilic residue Q in positions 58 and 67, respectively) and V62, which replaces A62 in OryR. These key differences suggest a different specificity toward most probably unrelated plant compounds for PsrR and OryR.

**FIG 5:**
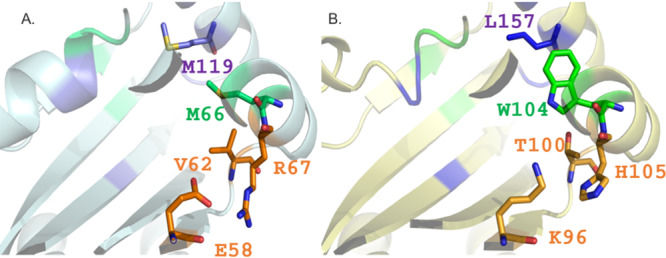
Comparison of the ligand-binding sites of PsrR and PipR. Shown is a superimposition of the 3D structure-based homology models of PsrR (A; colored in pale cyan) and PipR (B; colored in pale yellow), obtained from the orientation in Fig. 3 and 4 by 90° rotation around the *y* axis. Among the protein residues defining the three clusters (colored with the same color code as used in Fig. 2) that delineate the conserved core, the specificity patch, and the variability patch, the side chains of ones that are not conserved between PsrR and PipR are displayed. Structures were produced by PyMOL (73).

PipR is a recently reported PAB LuxR solo from *Pseudomonas* sp. strain GM79 and has been shown to respond to the plant compound *N*-(2-hydroxyethyl)-2-(2-hydroxyethylamino)acetamide (HEHEAA) (25). As PipR is the first PAB LuxR solo in which the inducing plant compound has been determined, it was of interest to compare its homology modeling to the one of PsrR. Figure 5 compares the ligand-binding sites of PsrR and PipR, highlighting the side chains of residues that are not conserved between PsrR and PipR. These residues define mainly the roof and part of the floor and of the proximal wall of the two binding pockets that are significantly different in terms of electrostatic potentials (E58 and R67 in PsrR instead of K96 and H105 in PipR) and hydrogen bond donor capabilities (V62 and M66 in PsrR instead of T100 and W104 in PipR) that may indicate different specificities of PsrR and PipR toward unrelated plant compounds.

### LoxR is promiscuous and binds to a wide spectrum of AHLs.

It was therefore of interest to determine if LoxR was able to bind AHLs due to its high homology to the SdiA three-dimensional (3D) structure and due to the conserved AHL binding docking residues (see above). His-tagged *loxR* was expressed in E. coli in the presence of different AHLs (C_6_-HSL, OHC_6_-HSL, OC_6_-HSL, OC_8_-HSL, and C_10_-HSL) by induction with 0.2 mM isopropyl-β-d-thiogalactopyranoside (IPTG) overnight at 37°C. Figure S1 in the supplemental material compares the total extracts to the soluble fractions; LoxR was soluble when bound and insoluble when unbound to AHLs, as was also reported for many QS LuxR family proteins (39). This result indicated that LoxR was promiscuous toward many different AHLs, similar to what previously reported for its SdiA homolog.

To further verify the promiscuity of LoxR, the expression protocol was further modified because of the low stability of LoxR, which was due to proteolytic cleavage in the linker resulting in two protein bands with molecular weights (MW) of the two domains. The optimization as described by Studier (40) allowed the expression of *loxR* in the presence of two very different AHLs (C_6_-HSL and OC_12_-HSL); OC_12_-HSL was also able to solubilize LoxR (data not shown) like the other 6 AHLs shown in Fig. S1. LoxR was then purified by nickel-nitrilotriacetic acid (Ni-NTA) affinity chromatography followed by gel filtration (Fig. S2). After optimization of expression levels employing the autoinduction protocol (40), it was possible to obtain similar amounts of Lox complexes with a yield of 3 mg liter^−1^. For confirmation of LoxR binding to AHLs, liquid chromatography-tandem mass spectrometry (LC-MS/MS) was performed for the protein eluted fractions corresponding to the putative LoxR-AHL complex (Fig. S3 and S4). In these cases, selective ion monitoring was used to identify the presence of the coeluting unlabeled AHL. The identity of the binding protein was confirmed by MS-based protein identification (Tables S1 and S2). It was therefore concluded that AHLs solubilize and bind to LoxR.

### LoxR and PsrR target gene studies.

**FIG 6:**
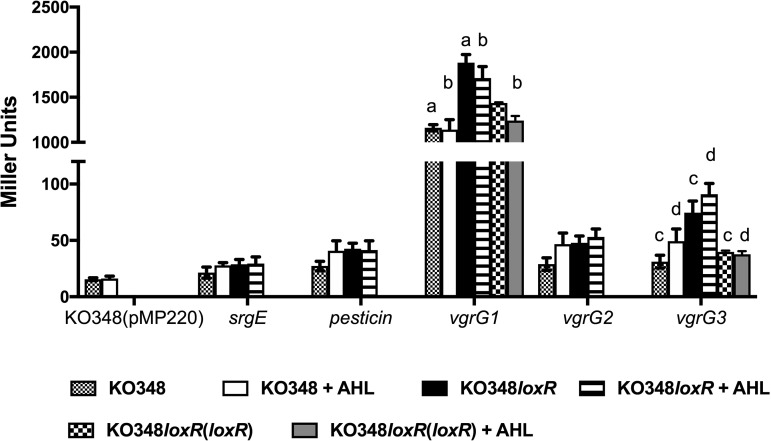
Gene promoter activities in the presence or absence of AHLs in *Kosakonia* KO348 and KO348 *loxR*. β-Galactosidase activities (Miller units) of five gene promoter transcriptional fusions (*srgE*, type VI cluster hypothetical protein named as pesticin, *vgrG1*, *vgrG2*, and *vgrG3* were tested) were determined to compare the expression levels between the WT (KO348), *loxR* mutant (KO348 *loxR*), and, when necessary, the complemented mutant KO348 *loxR*(pBBR*loxR*) in the presence or absence of AHLs. The WT strain with empty plasmid wt(pMP220) was used as control. All experiments were performed in triplicate. Statistical analysis was calculated using one-way ANOVA followed by Tukey’s multiple comparisons by Prism 7 (GraphPad Software, Inc.). Means with the same letter (a to d) indicate statistically different values (*P* < 0.05). The error bars indicate standard deviations.

It was of interest to test whether some loci known to be regulated by LoxR or PsrR homologs in other bacteria were regulated by the two LuxR solos in *Kosakonia*. In the case of LoxR, we studied the transcription driven by five gene promoters which have been reported to be regulated by SdiA (41). The loci regulated by these promoters were studied via transcriptional fusions with the *lacZ* reporter gene in a plasmid construct in *Kosakonia* KO348 and *loxR* mutant KO348 *loxR*. These gene promoters control the expression of the virulence-associated *srgE* gene and four type VI secretion system (T6SS)-related genes (one hypothetical gene and three *vgrG* genes). The T6SS has been shown to be regulated by SdiA in Enterobacter cloacae (41) and *srgE* in Salmonella enterica serovar Typhimurium. It was established that *srgE*, the hypothetical T6SS locus, and *vgrG2* were not regulated by LoxR in the presence or absence of AHLs (Fig. 6). The promoters of *vgrG1* and *vgrG3*, on the other hand, displayed differential expression in the presence and absence of AHLs in the KO348 *loxR* mutant in comparison to the wild type (WT) (Fig. 6). These differences were AHL independent and were only slight, albeit significant; hence, their meaning/implication remains to be determined. These little differences in gene promoter activities of *vgrG1* and *vgrG3* were restored to wild-type levels when the *loxR* mutant was complemented with a *loxR* gene in a plasmid (Fig. 6). Under the growth conditions used for the studies of gene promoter activities, no growth differences were observed between the KO348 wild type and all the derivatives (Fig. S5).

In the case of PsrR, since it belongs to the subfamily of PAB solos responding to plant low-molecular-weight molecules (15, 25), it was of interest to study the expression of the adjacent *pip* gene, encoding a proline iminopeptidase. All members of the subfamily of PAB LuxR solos have a *pip* gene located adjacently which is regulated by the LuxR solo (15). We tested *pip* promoter activity in the presence of (i) rice root macerate, (ii) ethanolamine, and (iii) ethanolamine derivative HEHEAA. The last two compounds were recently reported to activate *pip* expression via the PAB LuxR solo PipR in a *Pseudomonas* endophyte (25). No PsrR-dependent *pip* promoter activity was detected and no induction of *pip* was observed in *Kosakonia* under any of the conditions tested (Fig. S6A and B). Regardless of the fact that PsrR did not respond to plant macerate/extract, due to its very high homology to the PAB solos and proximity to the *pip* gene, it most likely responds to a plant-derived compound.

### Phylogenetic clustering of PAB LuxR solos.

**FIG 7:**
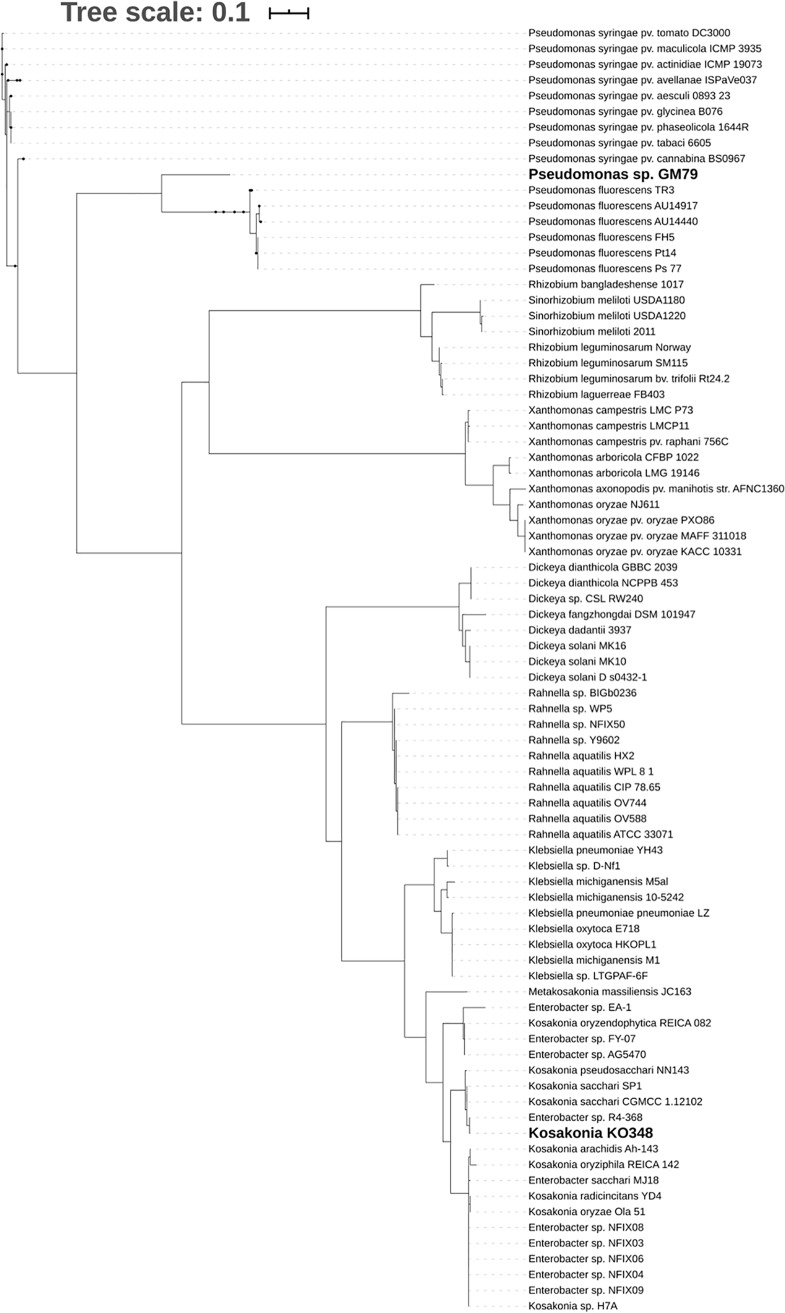
Phylogenetic tree showing the position of PsrR from *Kosakonia* KO348 with respect to other members of the LuxR PAB subfamily of solos.

In order to establish relatedness between PsrR and the PAB LuxR solos belonging to different plant-associated genera or species, a phylogenetic tree was created. LoxR is most closely related to other strains of *Kosakonia* and *Enterobacter* (data not shown). The tree of PAB LuxR solos belonging to many different plant-associated bacteria showed clustering of the proteins according to the genera that they belong to (Fig. 7). PsrR from *Kosakonia* and PipR from *Pseudomonas* GM79 were not closely related within this family, possibly suggesting that they might respond to related but different plant compounds, as supported in this study by the structural homology and *pip* gene promoter studies (Fig. 5; see also Fig. S6A and B in the supplemental material).

### Role of LoxR and PsrR in *in planta* studies.

**FIG 8:**
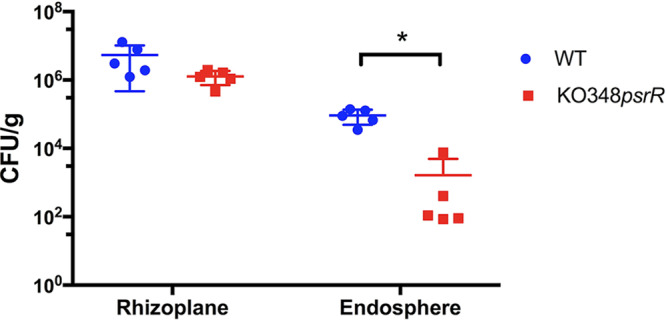
Role of PsrR solo of *Kosakonia* KO348 in rhizoplane and endosphere rice root colonization. The effect of PsrR was tested by comparing CFU per gram of WT KO348 versus the KO348 *psrR* mutant at rhizoplane and at endosphere of rice root plants at 14 days postinoculation. A Kruskall-Wallis test was used for specific pairs of data (WT KO348 versus KO348 *psrR*) by Prism 7 (GraphPad Software, Inc.).

*Kosakonia* strain KO348 is a very efficient rice root colonizer (35); thus, the role LoxR and PsrR LuxR solos play in colonization of the rhizoplane and root endosphere was studied. We generated *loxR* and *psrR* knockout mutants and determined rice rhizoplane and endosphere colonizing efficiencies. No statistically significant differences in colonization between the wild type and KO348 *loxR* were observed in the presence or absence of AHLs both in the rhizoplane and in the endosphere (Fig. S7). Similarly, no statistically significant difference in colonization of the rhizoplane between the wild type and KO348 *psrR* was observed. In the root endosphere, however, the wild type was a significantly better colonizer than the mutant KO348 *psrR* (Fig. 8), indicating the involvement of PsrR in the rice endosphere colonization of *Kosakonia* KO348.

## DISCUSSION

LuxR solos are very widespread in proteobacteria (8, 10, 23); however, only a few have been thoroughly studied. Here, two LuxR solos in the recently described genus *Kosakonia* are reported; LoxR is an SdiA homolog and is able to bind AHLs promiscuously, whereas PsrR belongs to the PAB subfamily that responds to plant compounds.

Modeling analysis of LoxR showed high conservation of amino acid residues forming the binding pocket just like in E. coli SdiA (PDB ID 4LGW), indicating that it is likely to bind AHLs. LoxR has conserved amino acids Y63, W67, Y71, D80, and S134 (according to SdiA numbering) (Fig. 2), which are important for the docking of different types of AHLs (42). Experimental evidence for LoxR binding to AHLs include protein solubilization in the presence of AHLs, which is likely necessary for proper folding and homodimerization (39), and detection of LoxR-AHL complexes in the purified recombinant protein. It is therefore most likely that LoxR detects and responds to a wide range of exogenous AHLs produced by neighboring bacteria, as is the case of SdiA from *Salmonella* and E. coli (19, 43). Promiscuity of LuxR receptors that respond broadly to AHL signals occurs also in QS LuxR proteins which possess cognate LuxI and hence can respond to non-self-signals (44). This property has implications in interspecies signaling, as bacteria are most commonly members of multispecies communities. SdiA is well conserved among members of the *Enterobacteriaceae* family; SdiA orthologs are present in *Escherichia*, *Kosakonia*, *Salmonella*, *Enterobacter*, *Citrobacter*, *Cronobacter*, *Klebsiella*, *Pantoea*, and *Erwinia* (45).

As SdiA is likely to be involved in interspecies signaling, identifying target loci will provide insights into its role in bacterial community lifestyle. In this study, some SdiA targets determined in other bacterial species were tested for LoxR regulation in *Kosakonia*. The *srgE* gene, encoding a type III effector protein, is regulated by SdiA in *Salmonella* (18, 19). The *srgE* gene, however, is not regulated by LoxR in *Kosakonia* under the conditions tested in this study, indicating that it could have evolved to regulate a different set of target genes. In E. cloacae, SdiA regulates expression of a hypothetical protein from the T6SS cluster (41); interestingly, we have determined that in *Kosakonia*, two T6SS *vgrG* loci are negatively regulated by LoxR, indicating that the T6SS might be a conserved SdiA target. In *Kosakonia* KO348, T6SS plays a role in rhizoplane and rice root endosphere colonization (35); thus, LoxR could play a role *in planta* by regulating genetic loci in response to AHLs produced by neighboring bacteria in the rhizosphere and endosphere. The performed *in planta* experiments do not corroborate this possibility; however, in the wild, growth conditions are different from the experimental setup used in this study, since *Kosakonia* is likely to coexist with many more different microbes (see below). SdiA homologs and the T6SS are commonly found in endophytic bacterial genomes; however, they are more prevalent among the genomes of phytopathogens (46). More studies are needed to understand the role and regulation of T6SS by SdiA homologs in plant-beneficial bacteria.

*In planta* studies performed in this investigation showed no involvement of LoxR in rhizoplane or endosphere colonization. In a PGP E. cloacae strain, however, an SdiA mutant displayed 4-fold more efficient rhizoplane colonization with respect to that of the wild type (47). The *in planta* experiment described here has some limitations since it lacked soil containing a rich and native microbial community, which is likely to undergo cell-cell communication, including AHL signaling. AHLs were added in the experimental setup; however, it is not known if they were stable and remained at high concentrations throughout the days of the experiment. Therefore, the role of SdiA in root colonization under wild/field conditions may not be inferred from the results presented here.

PsrR is another member of the subfamily of PAB LuxR solos which responds to plant compounds (15). Homology modeling was employed to compare the structural determinants of PsrR with two previously described PAB LuxR solo proteins, OryR from the rice pathogen Xanthomonas oryzae pv. oryzae (22) and PipR from endophytic *Pseudomonas* strain GM79 (48). PipR is the only member of this subfamily which has been shown to respond to a specific plant compound (25). Importantly, some key differences between PipR and PsrR were found. PsrR displays the two important amino acids, M and W (at positions W57 and Y61 according to TraR numbering), that are part of the autoinducer binding site; these amino acids are conserved among many PAB LuxR solo subfamily members (49) and are also shared between PsrR and OryR. However, at the same positions, PipR shows a W and W (at positions W57 and Y61 according to TraR numbering) (23). This indicates a key difference in the binding site residue conformation between PsrR and PipR, probably resulting in a different response/specificity to plant compounds. PipR activates *pip* target gene expression in response to an ethanolamine derivative, *N*-(2-hydroxyethyl)-2-(2-hydroxyethylamino)acetamide, at particularly low concentrations (25); such a response did not occur in *Kosakonia* KO348. It cannot be excluded that this assay does not work under the conditions tested due to the lack of a positive response by PsrR in the regulation of the *pip* gene. The PsrR primary structure has a difference in the binding pocket conformation in comparison to PipR, which possibly suggests different inducer specificity, supporting its likely nonresponsiveness to the PipR signal. This difference in specificity could be the reason for the distance in clustering observed in the phylogenetic tree (Fig. 7). This analysis evidences clustering according to the genus and indicates possible evolution of binding to different, possibly related plant compounds.

The PAB LuxR solos are believed to regulate in response to a plant compound; the adjacently located *pip* gene encodes a proline iminopeptidase that is believed to be involved in the signal response (48). We tested whether PsrR could respond to plant extracts and regulated *pip* gene expression. Rice root extract could not activate *pip* expression in root endophyte *Kosakonia* KO348; several *pip* genes have, however, been shown to be activated by plant extract via PAB LuxR solos, as, for example OryR, PipR, and XccR (24, 48, 50), whereas some could not (49). The reason for this lack of response by plant extract by PsrR is currently unknown; it cannot be excluded that the signal molecule is present in very low concentrations or only in specific plant parts and/or growth stages. Alternatively, *psrR* could be itself regulated and under these experimental conditions the expression level is too low. In the promoter of the adjacent *pip* genes, a DNA-binding region resembling a *lux* box has been reported for several members of this LuxR solo subfamily (22). In this case, a DNA region resembling a *lux* box (or *pip* box) is present at the region from −19 to −39 upstream of the ATG start codon of the *pip* gene (CCCTGAAAGCCAGTTCACTT) and −176 to 196 of the *psrR* gene (CCCTGGTTATCCGCGCCCGG). These findings could indicate regulation and autoregulation by PsrR, respectively; future research is needed to confirm the possible functionalities of these putative DNA-binding sites. It is also noted that the intergenic region between *psrR* and *pip* (Fig. 1B) is only 84 bp; hence, we cannot exclude an operonic structure between *psrR* and *pip*.

*In planta* studies indicated that PsrR is involved in root endosphere colonization, since the mutant displays significantly less colonization. This phenotype could not, however, be complemented when providing the wild-type gene in a plasmid; a possible explanation could be the high percentage of plasmid loss in the endosphere, as previously reported for *Kosakonia* (35). Other PAB LuxR solos have been implicated in plant colonization, for example, PsoR of Pseudomonas fluorescens, XocR of *X. oryzae*, PsaR2 of Pseudomonas syringae pv. actinidiae, and XagR of Xanthomonas axonopodis pv. glycines (21, 23, 49, 51).

This study further highlights the presence of LuxR solos in proteobacteria, which are likely to play important roles in cell-cell signaling. These have most likely evolved from *luxR* genes which were part of a *luxI/R* system (45) and some have then evolved to respond to other endogenous or exogenous signals. A future challenge will be to identify the signals that they respond to.

## MATERIALS AND METHODS

### Bacterial strains and growth conditions.

*Kosakonia* KO348 was previously isolated from the root endosphere from rice grown in Italy (34); its genome was published in DDBJ/EMBL/GenBank (accession no. JZLI00000000) (36). KO348, KO348 *loxR*, KO348 *psrR*, and Escherichia coli strains DH5α, S17, and BL21(DE3) were routinely grown at 30°C and 37°C in Luria-Bertani (LB) broth medium (52) or in MME minimal medium (35). When required, antibiotics for *Kosakonia* strain growth were added at the following concentrations: rifampin, 50 μg ml ^−1^; gentamicin, 25 μg ml^−1^; and kanamycin, 100 μg ml^−1^. Antibiotics for E. coli were added at the following concentrations: ampicillin, 100 μg ml^−1^, and gentamicin, 15 μg ml^−1^. *N*-Hexanoylhomoserine lactones (C_6_) and *N*-3-oxododecanoyl-homoserine lactone (OC_12_-HSL) were obtained from Sigma-Aldrich (St. Louis, MO).

### Structure homology modeling.

The structure-based sequence alignment of the ligand-binding domains of LoxR and PsrR was performed using the program Expresso (53) (Fig. 2). The following primary sequences were included in the multiple alignment: PipR from *Pseudomonas* sp. GM79 and OryR from Xanthomonas oryzae, prototypes of the PAB LuxR solo subfamily, and TraR from Agrobacterium tumefaciens TraR_*At* (PDB ID 1H0M) (54) and from Sinorhizobium fredii NGR234 TraR_*Sf* (PDB ID 2Q0O) (55) prototypes of the canonical QS LuxR proteins. The templates used in the structure-based homology modeling of LoxR and PsrR were SdiA from Escherichia coli (PDB ID 4LGW) (56) and QscR (PDB ID 3SZT) (57) and LasR (58) from P. aeruginosa. Structure-based homology modeling of full-length LoxR and PsrR was performed exploiting several approaches, and the resulting models were ranked according to protein model quality assessments based on LG score and MaxSub. In order to elucidate the molecular determinants of ligand discrimination in LoxR and PsrR, a cartographic analysis of the top-ranked models has been exploited as described in reference 37.

Several web-based servers were used to build the 3D structure-based homology models of full-length LoxR, PsrR, and PipR. The top-score models generated by the servers were then ranked and validated by the protein model quality predictor ProQ (59), employing PSIPRED (60) for the secondary-structure prediction. The IntFOLD server (61) produced the highest-quality 3D models for LoxR and PsrR from *Kosakonia*, according to the ranking obtained by ProQ, i.e., a predicted LG score and MaxSub value of 4.749 and 0.486, respectively, for LoxR and 4.125 and 0.706, respectively, for PsrR. The protein model of PipR was obtained by RaptorX (62), with a predicted LG score and MaxSub value of 4.039 and 1.010, respectively.

The templates used for LoxR modeling were SdiA from E. coli (PDB ID 4LGW [56]) and QscR from P. aeruginosa (PDB ID 3SZT [57]). The PsrR model was obtained using the same templates as for LoxR combined with LasR (PDB ID 3IX3 [58]) from P. aeruginosa. The template used for PipR modeling was QscR from P. aeruginosa (PDB ID 3SZT [57]).

### Protein production and purification.

The *loxR* gene was amplified from *Kosakonia* KO348 using primers lox_Fw (CATATGCAGGATACAGAATTCTTTACC) and lox_Rev (ACTCGAGAATCATCCCTGTCGCCGCTGC) and directionally cloned at the NdeI/XhoI restriction sites into His_6_-tagged protein expression vector pET22b (Addgene, Watertown, MA) which was later transformed in E. coli. The *loxR* expression levels were optimized by small-scale expression testing using several E. coli strains, and BL21(DE3) pLysS was found to be the most efficient one. For large-scale expression, *loxR* was expressed by autoinduction following previously described (40) methodology at 16°C overnight in the presence of 10 μM AHLs (C_6_-HSL or OC_12_-HSL). Cells were harvested, suspended in lysis buffer (50 mM Tris base buffer [pH 8.5], 300 mM NaCl, 1 mM EDTA, 5 mM imidazole, 5 mM β-mercaptoethanol, 5% glycerol), and lysed by sonication. After 1 h of centrifugation at 10,000 rpm, the filtered lysates were loaded on a 5-ml HisTrap fast-flow (FF) crude column (GE Healthcare Inc., Chicago, IL). The column was washed with wash buffer (50 mM Tris base buffer [pH 8.5], 300 mM NaCl, 0.1 mM EDTA, 10 mM imidazole, 5 mM β-mercaptoethanol, 5% glycerol) and eluted in a linear gradient with elution buffer (50 mM Tris base buffer [pH 8.5], 300 mM NaCl, 0.1 mM EDTA, 250 mM imidazole, 5 mM β-mercaptoethanol, 10% glycerol). The eluted fractions were collected for SDS-PAGE analysis, pooled, and concentrated by VivaSpin (GE Healthcare Inc.) for size exclusion chromatography in 50 mM Tris base buffer (pH 8.5; 300 mM NaCl, 0.1 mM EDTA, 5 mM β-mercaptoethanol, 10% glycerol). The complex with C_6_-HSL was purified by four runs on Superdex 75 10/300 GL (GE Healthcare), while the complex with OC_12_-HSL was loaded on a HiLoad 200 16/60 column (GE Healthcare). Peak fractions were pooled, concentrated, flash frozen, and stored at –80°C.

### Mass spectrometry.

Aliquots of the peak fractions described above were denatured by heating to 95°C for 5 min and then subjected to reduction, alkylation, and digestion with trypsin. After digestion, they were dried down and resuspended in 20 μl of 0.1% formic acid. LC-MS/MS of the digestion was performed using an Easy-nLC II coupled to an Amazon ETD mass spectrometer (Bruker Daltonics, Hamburg, Germany). The same digests were used to screen for the presence of AHLs. The elution times and fragmentation behavior of the AHLs were scouted using purified standards (C_6_-HSL and OC_12_-HSL; Sigma-Aldrich Inc., St. Louis, MO). Protein identification was performed using the X!tandem (www.thegpm.org) search engine and a *Kosakonia* KO348 database.

### Construction of KO348 *loxR* and KO348 *psrR*.

To generate KO348 *loxR*, an internal fragment (312 bp) of the *loxR* gene was amplified by PCR using the primers pKNloxR.Fw (GATGCAGCATTATCAGGCAGA) and pKNloxR.Rv (TGATCTTCCAGACGCGTTAA) and cloned as a BamHI-KpnI fragment into the corresponding sites of pKNOCK-Km (63), resulting in pKNOCKloxR. To generate KO348 *psrR*, an internal fragment (245 bp) of the *psrR* gene was amplified by PCR using the primers pKNpsrR.Fw (CTATCAACGCCCGGACAG) and pKNpsrR.Rv (ACAGCGGAAAGGCAGATT) and cloned as a BamHI-KpnI fragment into the corresponding sites of pKNOCK-Km, resulting in pKNOCKpsrR. Plasmid constructs were delivered to *Kosakonia* KO348 by electroporation, and transformants were selected after appropriate antibiotic selection. The *loxR and psrR* full-length genes (including their gene promoters) were amplified with the primers LoxR-comp_Fw (GGATCCCCGTTAACGTTGGCGTTAAA) and LoxR-comp_Rv (GAATTCTTTAAATCATCCCTGTCGCC) for the *loxR* gene and PsrR-comp_Fw (GGATCCACGGTGCATCAGCATTCTCC) and PsrR-comp_Rv (GAATTCGGCGCTGAACACTAGCAAAA) for the *psrR* gene; the sequences were verified via DNA sequencing, and the resulting fragments were cloned into the pBBR1MCS-5 vector (64). The plasmids were electroporated in the mutant strains KO348 *loxR* and KO348 *psrR*, respectively, and selected for Km^r^ and Gm^r^; the resulting mutant complemented strains were named KO348 *loxR*(pBBR*loxR*) and KO348 *psrR*(pBBR*psrR*). Mutants and complemented mutants were verified by colony PCR and DNA sequencing.

### Gene promoter studies.

Transcriptional activity studies of six gene promoters (*srgE*, hypothetical T6SS gene, three different *vgrG* genes, and the *pip* gene promoter) were performed with *Kosakonia*. Gene transcriptional fusion plasmids were constructed in the pMP220 promoter probe vector, which harbors a promoterless *lacZ* gene (65). The primers used for the cloning of the gene promoters were as follows: promsrgE_FwBam (GGATCCGCTTGGACAGGATTGTTTATTG) and promsrgE_RvEco (GAATTCTTCCCTGTTCCTTAGCGTGT) (*srgE*PROM; 345 bp), promphyp_FwBam (GGATCCGGCGGTAAAGGTGCTGAA) and promhyp_RvEco (GAATTCATCACCCTTGCGCATAAC) (PROMhypprot; 287 bp), promVgrG1_FwBglII (AGATCTCGCGTGAAAGTGGGATAATA) and promVgrG1_RvEco (GAATTCCATCGACGGGTAACTGAAC) (*vgrG1*PROM; 381 bp), promVgrG2_FwBam (GGATCCGCCAAGCCAGTTCAAAGTA) and promVgrG2_RvEco (GAATTCCCCGGAGAGTTTCCAGA) (*vgrG2*PROM; 200 bp), promVgrG3_FwBam (GGATCCAGGCACCTGGCTTATCA) and promVgrG3_RvEco (GAATTCCCCTGATTGCTGTGTGTT) (*vgrG3*PROM; 205 bp), and prompip_FwBam (GGATCCGCGTTGCACTACCGTCT) and prompip_RvEco (GAATTCGAAGGGAACGTAGCCTT) (pipPROM; 146 bp). After PCR amplification using genomic DNA as the template, and verification of the fidelity by DNA sequencing, the corresponding fragments were digested using BamHI and EcoRI and cloned into the corresponding sites in promoter vector pMP220.

β-Galactosidase activity was determined as previously described (52), with the modifications described in reference 66. Promoter activities were performed in biological triplicates at the onset of stationary phase. When necessary, two AHLs (C_6_-HSL and OC_12_-HSL) were added each at a concentration of 1 μM. The *pip* gene promoter activity was also determined in the presence of rice root extract, ethanolamine (500 μM) obtained from Sigma-Aldrich (St. Louis, MO), and *N*-(2-hydroxyethyl)-2-(2-hydroxyethylamino)acetamide (HEHEAA; 10 μM) obtained from Chiron AS (Trondheim, Norway).

### Phylogenetic tree construction for PsrR.

The amino acid sequence of *Kosakonia* KO48 PsrR solo was searched by BLAST against the genomes available in the U.S. Department of Energy IGM/M database, and the top homologs were identified. Representative homologs from the genera with previously described LuxR plant-associated solo subfamily sequences were chosen to be displayed phylogenetically. The sequences were aligned using Clustal Omega standard settings with FASTA-formatted output. The alignment was trimmed using TrimAI v1.3 using the “automated1” flag. The trimmed alignment was put into Fasttree 2.1.10 using standard settings. The tree was visualized on Interactive Tree of Life (67–71).

### Rice root colonization assays.

Rice root colonization experiments were performed as described previously (34), with the following modifications. Briefly, each strain was grown to an optical density at 600 nm (OD_600_) of 0.8 and used to inoculate 9-day-old germinated rice plant roots (cultivar Baldo) by submerging them in the bacterial suspension for 1 h. Plants were then transferred to a 50-ml tube containing Hoagland’s semisolid solution (72); when necessary, AHLs were added at a concentration of 1 μM. All plants were followed for 14 days and then the *Kosakonia* strain was reisolated.

For rhizoplane colonization, rice roots were rinsed with sterile water for removing all adhered Hoagland’s solution and then vortexed in 5 ml of phosphate-buffered saline (PBS) solution for 1 min. Serial dilutions of this PBS solution containing bacteria were then plated on the appropriate media with antibiotics for calculation of CFU per gram. For the determination of endosphere colonization, the roots were sterilized as described previously (34), macerated in PBS, and then plated after serial dilutions in Trypticase soy agar (TSA) containing the appropriate antibiotics; the plates were then incubated at 30°C for 48 h and CFU per gram were calculated. For comparing the rhizoplane and endosphere colonization ability, analysis of variance (ANOVA) was performed with Prism 7 (GraphPad Software, Inc.). Five biological replicates were used in each group.

## Supplementary Material

Supplemental file 1
