# Supplementary material for: LuxR Solos in the Plant Endophyte Kosakonia sp. Strain KO348
Source: Appl Environ Microbiol. 2020 Jun 17;86(13):e00622-20. doi: 10.1128/AEM.00622-20 (PMC7301841; doi:10.1128/AEM.00622-20)
Supplement: Supplemental file 1 [file AEM.00622-20-s0001.pdf]

LuxR solos in a plant endophytic *Kosakonia*

Susan Mosquito[1], Xianfa Meng[1], Giulia Devescovi[1], Iris Bertani[1], Alex Geller[2], Asaf Levy[2], Michael P Myers[1], Cristina Bez[1], Sonia Covaceuszach[3] and Vittorio Venturi[1*]

Supplementary Figures

Supplementary Figure 1. SDS-PAGE 12% gels showing the location of overexpressed LoxR in the total extracts (A) and soluble fractions of the homogenates (B). Lanes 1, without IPTG; lanes 2, induced by IPTG without AHLs; lanes 3, induced in presence of C6-HSL; lanes 4, induced in presence of OHC6-HSL; lanes 5, induced in presence of OC6-HSL; lanes 6, induced in presence of OC8-HSL; lanes 7 induced in presence of C10-HSL. The bands corresponding to the full-length protein are highlighted by a star and the bands resulting from proteolysis by an arrow.

Supplementary Figure 2. Purification of LoxR in complex with C6-HSL (A, C) and with OC12-HSL (B, D): A, B: Ni-NTA affinity purification-imidazole gradient (green line 10-250 mM) in FPLC Akta (GE Healthcare). C, Size Exclusion in Superdex 75 10/300 GL column in FPLC Akta (GE Healthcare). Blue line: UV measure (mAU). D, Size Exclusion in HiLoad 16/60 Superdex 200 column in FPLC Akta (GE Healthcare). Blue line: UV measure (mAU). The inserts report the SDS-PAGE gels of the fractions corresponding to the elution peaks.

Supplementary figure 3. Binding of C6-HSL to LoxR using LC-MS/MS. LoxR was digested with trypsin, dried down and resuspended in 20 μl of 0.1% Formic acid. 2 μl were injected onto the LC-MS/MS system and ions with a m/z of 200.1 were subjected to fragmentation (top). The arrow shows the elution point of C6-HSL and the lower panel shows the fragmentation spectra of this peak. Fragmentation of C6-HSL (bottom) gives rise to two principle ions: 102 which is found in all AHLs and 182 which is specific to C6 AHL.

Supplementary figure 4. Binding of OC12-HSL to LoxR using LC-MS/MS. LoxR was digested with trypsin, dried down and resuspended in 20 μl of 0.1% Formic acid. 2 μl were injected onto the LC-MS/MS system and ions with a m/z of 298.2 were subjected to fragmentation (top). The arrow shows the elution point of OC12-HSL and the lower panel shows the fragmentation spectra of this peak. Fragmentation of OC12 (bottom) gives rise to two principle ions: 102 which is found in all AHLs and 197 which is specific to OC12-HSL.

Supplementary figure 5. Growth curves of KO348 and derivatives. *Kosakonia* wild type strain KO348 and derivatives were grown in liquid medium in biological triplicates and their growth monitored through a period of time as indicated. All strains grew at comparable levels and no significant differences were observed.

Supplementary figure 6. (A) *Kosakonia* KO348 *pip* promoter activity is not influenced by ethanolamine (a) or its derivative HEHEAA (b) β-galactosidase activities (Miller Units) determined for the *pip* promoter transcriptional fusion (*pip*) and KO348 WT containing the empty plasmid KO348(pMP220) in LB or

MME media supplemented with either ethanolamine or HEHEAA. All experiments were performed in triplicate. **(B)** *Kosakonia* KO348 *pip* promoter activity in presence of different concentrations of rice root extract**.** β-galactosidase activities (Miller Units) determined for the *pip* promoter transcriptional fusion (*pip*) and KO348 WT containing the empty plasmid KO348(pMP220) grown in LB or MME media supplemented with rice root extract.

Supplementary figure 7. Role of LoxR solo of *Kosakonia* KO348 in rhizoplane and endosphere rice root colonization. The effect of LoxR was tested by comparing CFU/g of KO348 WT vs. KO348*loxR* mutant in the rhizoplane (**A**) and in the endosphere (**B**) of rice root plants at 14 dpi in presence or absence of *N*-acyl-homoserine lactones (AHL).

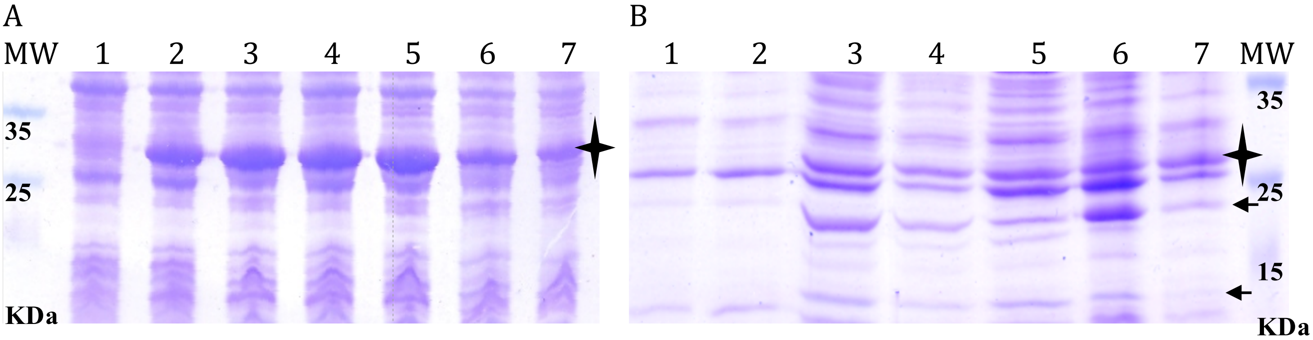

Supplementary figure 1A and 1B

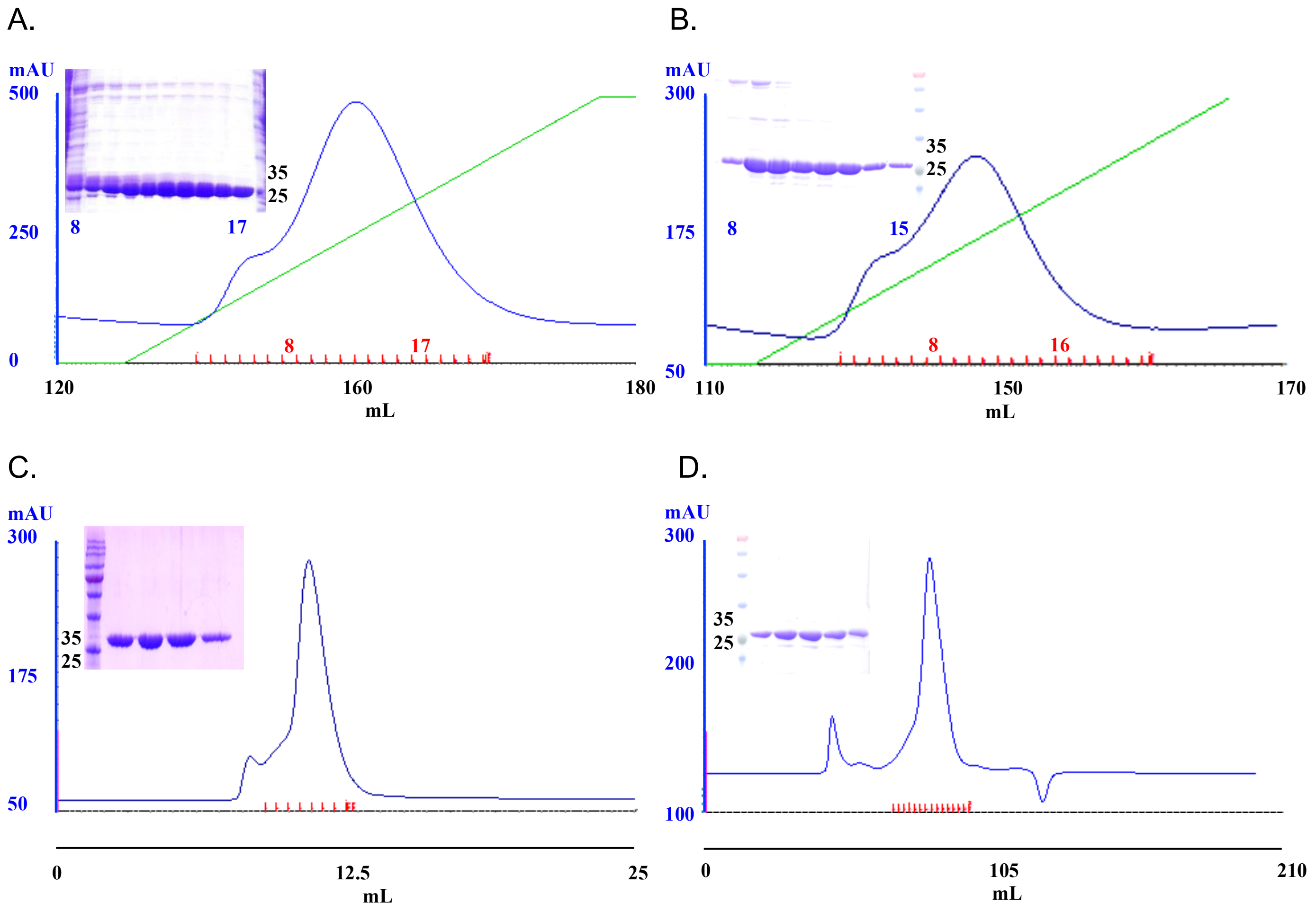

Supplementary figure 2A, 2B, 2C and 2D

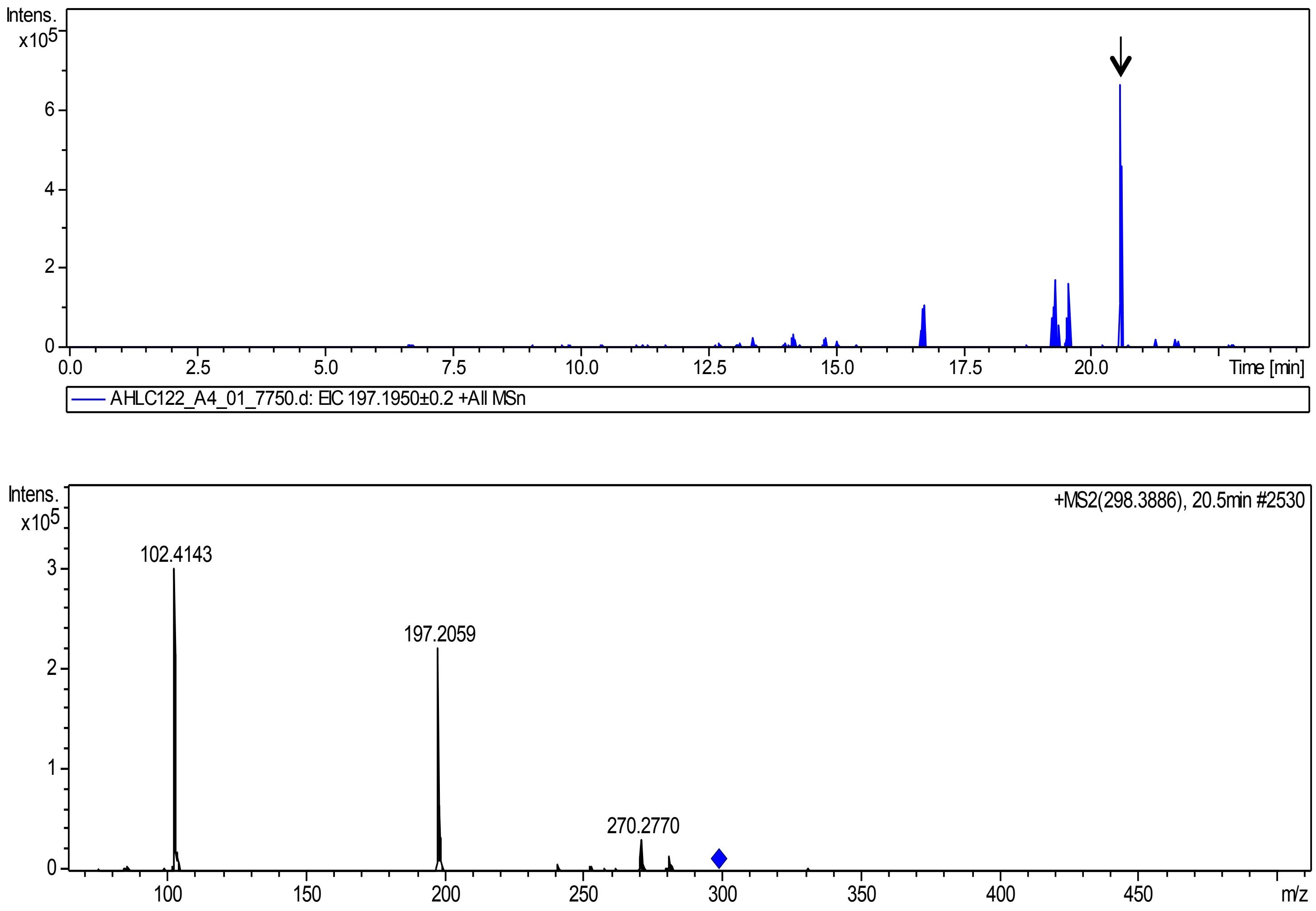

Supplementary figure 3

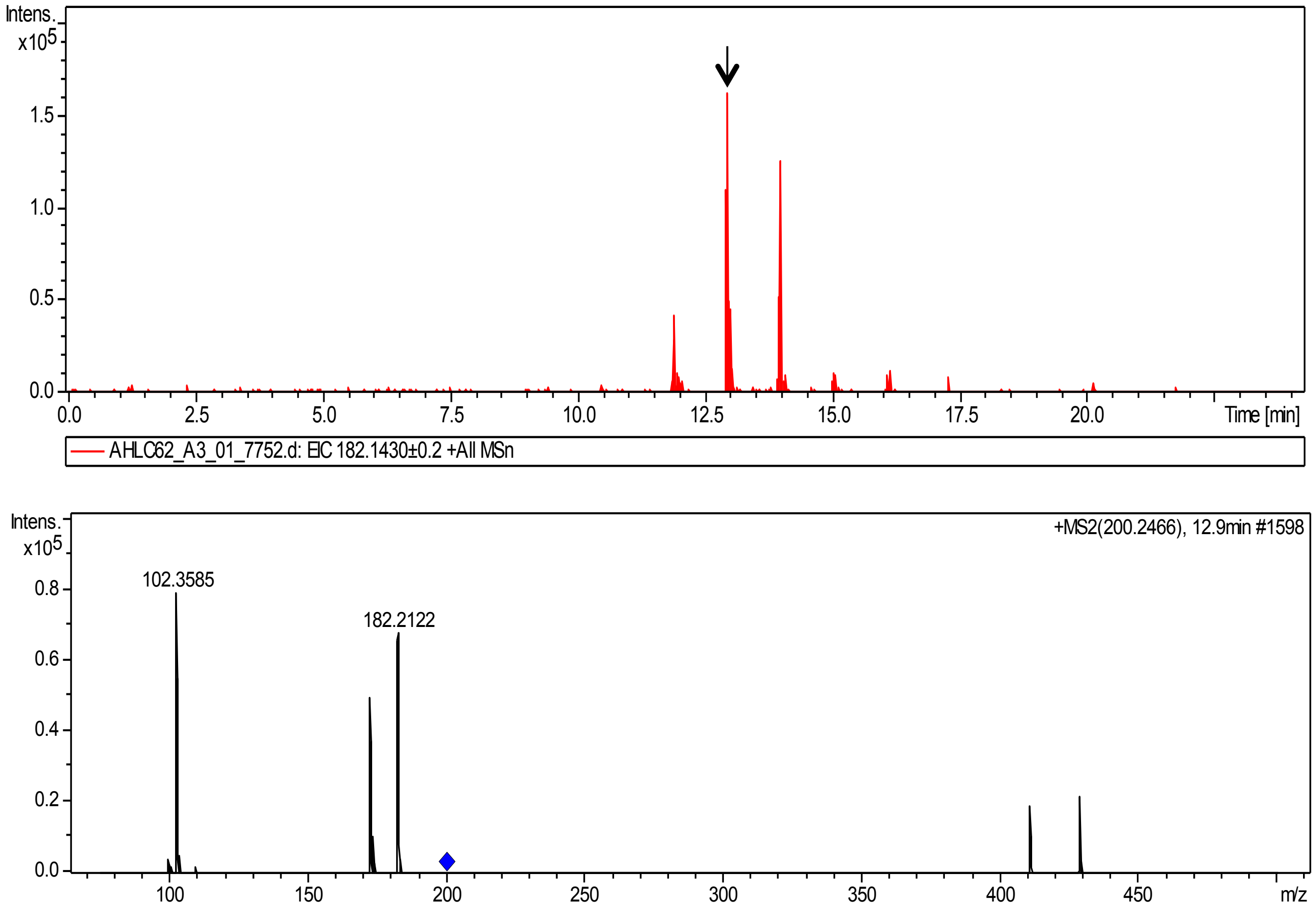

Supplementary figure 4

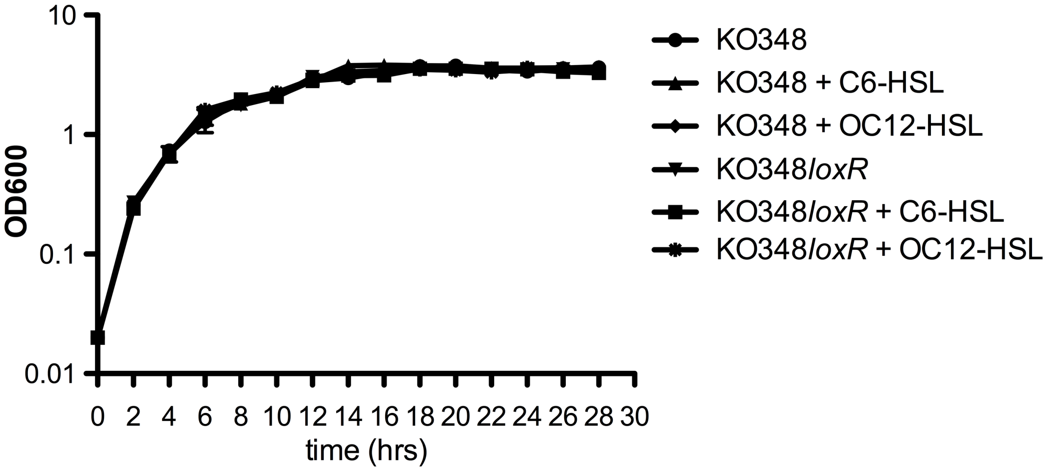

Supplementary figure 5

A.

a.-

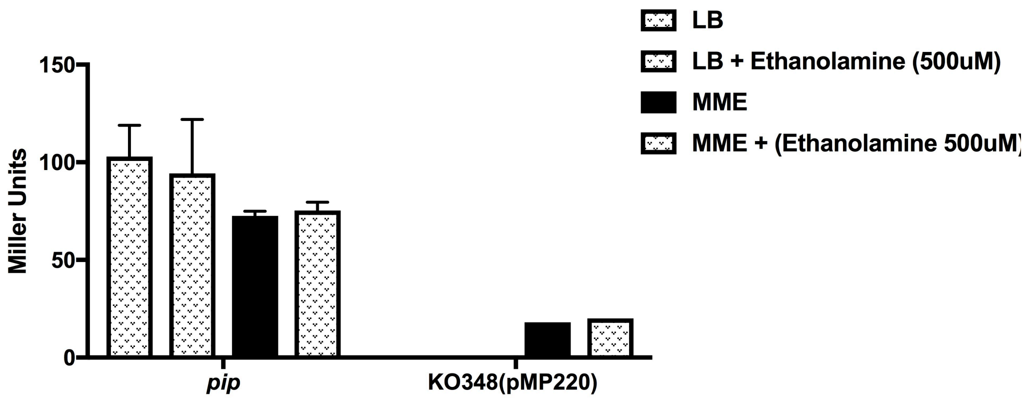

b.-

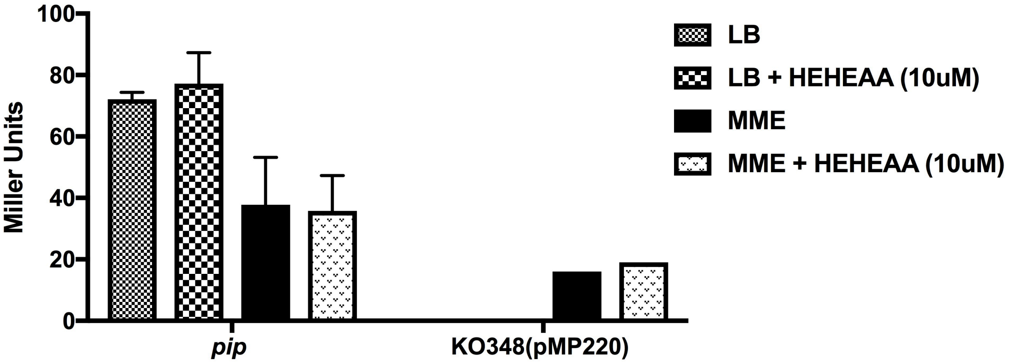

Supplementary figure 6A

B.

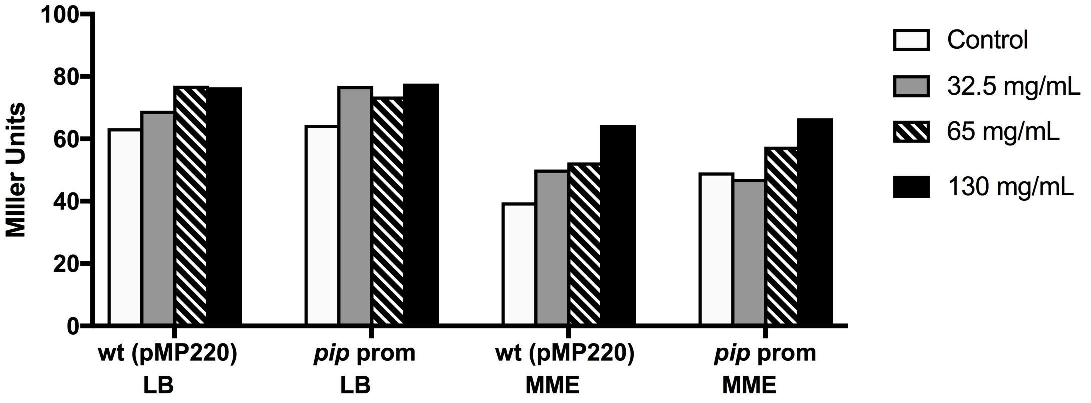

Supplementary figure 6B

a.-

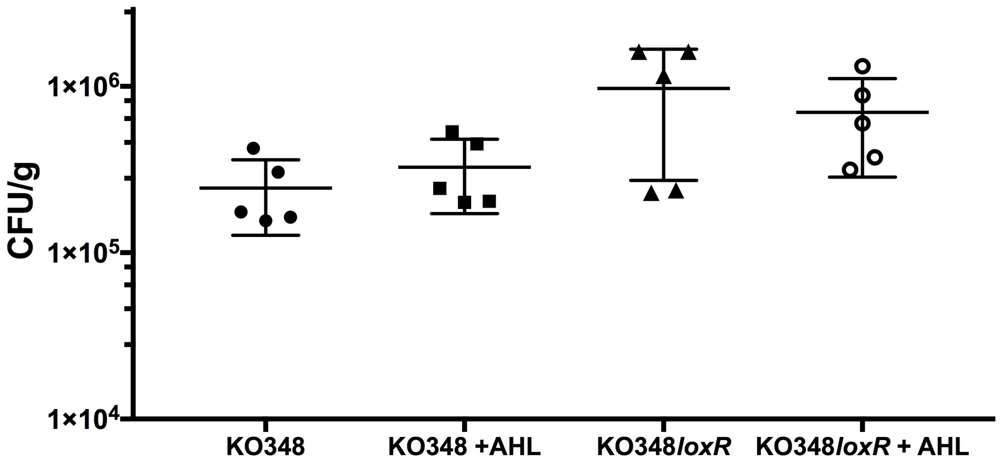

b.-

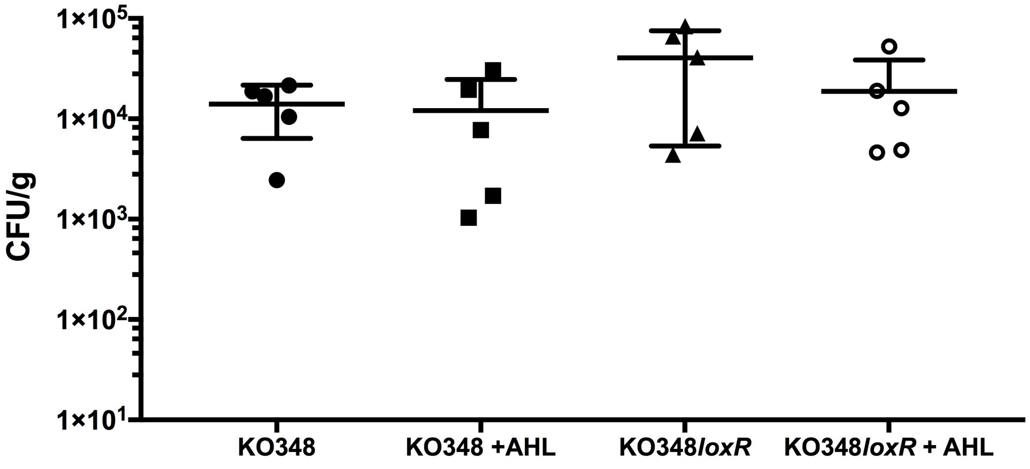

Supplementary figure 7
